# Use of Saliva Swab for Detection of Influenza Virus in Patients Admitted to an Emergency Department

**DOI:** 10.1128/spectrum.00336-21

**Published:** 2021-08-25

**Authors:** Alicia Galar, Pilar Catalán, Lara Vesperinas, Iria Miguens, Ioana Muñoz, Alejandro García-Espona, José Antonio Sevillano, Juan Antonio Andueza, Emilio Bouza, Patricia Muñoz

**Affiliations:** a Clinical Microbiology and Infectious Diseases Department, Hospital General Universitario Gregorio Marañón, Madrid, Spain; b Gregorio Marañón Health Research Institute, Madrid, Spain; c Emergency Department, Hospital General Universitario Gregorio Marañón, Madrid, Spain; d CIBER de Enfermedades Respiratorias (CIBERES CB06/06/0058), Madrid, Spain; e Medicine Department. School of Medicine, Universidad Complutense de Madrid, Madrid, Spain; Memorial Sloan Kettering Cancer Center

**Keywords:** influenza, saliva swab, nasopharyngeal swab, emergency department, diagnosis

## Abstract

Nasopharyngeal (NP) specimens are commonly used for the detection of influenza, but saliva swabs are easier to obtain and cause less discomfort to the patients. The objective of this study was to evaluate the usefulness of saliva swab specimens for the diagnosis of influenza compared with NP specimens. Influenza virus detection rate in saliva and NP swabs was compared in adult patients admitted to an emergency department from January to March 2020, using the Xpert Xpress Flu/respiratory syncytial virus (RSV) test. Cycle threshold (*C_T_*) values were evaluated in all the cases. Among the 82 patients recruited, 19 had an influenza-positive diagnostic test result (11 influenza A and 8 influenza B). Overall, the agreement between saliva and NP swabs results was 97.6% (80/82; κ = 0.929; 95% confidence interval [CI], 0.832 to 1.0). There was no significant difference in the influenza detection rate between saliva swab and NP specimens (20.7% [17/82] versus 23.2% [19/82]; *P* = 0.5). There were only two discordant results (influenza B in an NP and false negative in a saliva sample). Manual inspection of the amplification curves showed that influenza RNA had been amplified in saliva with high *C_T_*s (*C_T_* of 40) that the test reported as a negative result. The overall sensitivity and specificity for saliva was 89.5% (73.0% to 100%) and 100% (99.2% to 100%), respectively. In all the cases, the same influenza virus (A/B) was detected. Median *C_T_* values were significantly lower in NP (31; interquartile range [IQR], 21.0 to 32.0) than in saliva (33; IQR, 23.0 to 38.0) (*P* = 0.001) specimens. Saliva swabs have high sensitivity and specificity for the detection of influenza virus by the Xpert Xpress Flu/RSV test and a high overall agreement and *C_T_* correlation with NP specimens. Saliva swab is a feasible specimen type for influenza testing that might be easily self-collected with minimal equipment and discomfort.

**IMPORTANCE** Early detection of influenza virus is important for guiding antiviral and antibacterial treatment for infection control and public health measures. We have observed that saliva swab specimens have high sensitivity and specificity for the detection of influenza by the Xpert Xpress Flu/respiratory syncytial virus (RSV) test and high overall agreement and *C_T_* correlation with nasopharyngeal specimens. Saliva swab may therefore be a feasible specimen type for influenza testing that can be easily self-collected with minimal equipment and discomfort.

## INTRODUCTION

Influenza virus infection is a global health problem that, according to the World Health Organization, affects 10% to 15% of the world’s population, causing 600 million cases, 3 million serious illnesses, and 500,000 deaths per year.

Influenza virus diagnosis might sometimes be delayed because an adequate sample has not been obtained, which is essential for early clinical management, appropriate use of antimicrobials, and patient isolation (1). Currently, the microbiological diagnosis of influenza infection is simple and usually based on the performance of reverse transcriptase PCR (RT-PCR) of a respiratory sample (2–5). Nasopharyngeal (NP) exudate is the most commonly used sample type, which is not always easy to obtain with optimal quality. Sending NP samples from uncooperative patients or samples obtained by inexperienced personnel may lead to nosocomial spread of respiratory viruses, diagnostic delays, and false-negative results (6).

The utility and yield of saliva samples for the diagnosis of respiratory viruses, such as influenza and severe acute respiratory syndrome coronavirus 2 (SARS-CoV-2), are controversial (7–12). The collection of large volumes of saliva is usually recommended, but this is frequently a cumbersome procedure. In our experience, many patients are not able to spit out the sufficient volume of saliva needed for the detection of RNA virus with the current molecular techniques. Nevertheless, a saliva swab is easier to obtain and causes less discomfort in the patients, and its use might be a faster and much more convenient for influenza diagnosis than conventional saliva collection and NP swab.

The purpose of this study was to determine the diagnostic utility of influenza RT-PCR of a saliva swab specimen compared with the conventional NP exudate during the influenza season and verify whether the saliva swab could replace or complement the NP exudate in the diagnosis of influenza in adults in an emergency department.

(Part of this work was presented at the European Congress of Clinical Microbiology and Infectious Diseases [ECCMID] held virtually in July 2021.)

## RESULTS

### Patient characteristics.

From January to March 2020, a total of 85 patients were screened and 82 were eligible for the study (Table 1). The median age was 72 years old and 46.3% (38/82) were male. Heart disease was the most common underlying disease (39%, 32/82), followed by chronic obstructive pulmonary disease (COPD) (34.1%, 28/82) and diabetes mellitus (20.7%; 17/82). Thirty-nine patients (47.6%, 39/82) had received a vaccine against influenza virus and 30 (36.3%, 30/82) were immunosuppressed.

**TABLE 1:** Characteristics of patients with suspected influenza infection^*a*^

Characteristic	Data^*b*^ by category:			*P* value
	Global (*n* = 82)	Influenza (*n* = 19)	No influenza (*n* = 63)	
Demographics and clinical data				
Age (median [IQR])	72 (53.0–84.2)	62.0 (49.0–84.0)	74 (54.0–85.0)	0.529
Sex, male	38 (46.3)	8 (42.1)	30 (47.6)	0.795
Hospital stay, days (median [IQR])	1 (0–6)	2 (0–6)	1 (0–6)	0.648
Flu vaccinated	39 (47.6)	10 (52.6)	29 (46.0)	0.794
Immunosuppression	30 (36.6)	5 (26.3)	25 (39.7)	
Prednisone/corticoid equivalent of ≥15 mg/day for ≥30 days	9 (11.0)	1 (5.3)	8 (12.7)	0.452
Solid organ transplant recipient	6 (7.3)	1 (5.3)	5 (7.9)	1.0
Hematological transplant recipient	3 (3.7)	0	3 (4.8)	0.581
HIV with <200 CD4	3 (3.7)	2 (10.5)	1 (1.6)	0.133
Other	9 (11.0)	1 (5.3)	8 (12.7)	0.452
Symptoms				
Cough	50 (60.9)	12 (63.2)	38 (60.3)	1.0
Fever	48 (58.5)	12 (63.2)	36 (57.1)	0.792
Dyspnea	27 (32.9)	6 (31.6)	21 (33.3)	1.0
Auscultation abnormalities	15 (18.3)	3 (15.8)	12 (19.0)	1.0
Chest pain	8 (9.8)	1 (5.3)	7 (11.1)	0.674
Sputum	6 (7.3)	3 (15.8)	3 (4.8)	0.134
Headache	3 (3.7)	1 (5.3)	2 (3.1)	1.0
Rhinitis	3 (3.7)	0	3 (4.8)	0.581
Wheezing	1 (1.2)	0	1 (1.6)	1.0
Pharyngitis	0	0	0	NA
Underlying diseases				
Heart disease	32 (39.0)	9 (47.4)	23 (36.5)	0.430
COPD	28 (34.1)	8 (42.1)	20 (31.7)	0.420
Diabetes	17 (20.7)	5 (26.3)	12 (19.0)	0.526
Solid tumor	8 (9.8)	1 (5.2)	7 (11.1)	0.674
Chronic renal disease	7 (8.5)	1 (5.3)	6 (9.5)	0.684
Hypertension	5 (6.1)	0	5 (7.9)	0.336
Liver disease	4 (4.9)	2 (10.5)	2 (3.1)	0.228
Malignant/hematological neoplasm	4 (4.9)	0	4 (6.3)	0.569
HIV	3 (3.7)	2 (10.5)	1 (1.6)	0.133
Neurologic disease	3 (3.7)	1 (5.3)	2 (3.1)	1.0
Autoimmune disease	3 (3.7)	1 (5.3)	2 (3.1)	1.0
Psychiatric illness	3 (3.7)	0	3 (4.8)	0.581
Hemodialysis	1 (1.2)	1 (5.2)	0	0.232
Solid organ transplant recipient	0	0	0	NA
Pregnancy	0	0	0	NA
Charlson’s comorbidity score, age adjusted	6 (1-9.0)	7 (0-10.0)	6 (2-8)	0.744
McCabe and Jackson score				0.871
Nonfatal	56 (68.3)	14 (73.7)	42 (66.7)	
Ultimately fatal	24 (29.3)	5 (26.3)	19 (30.2)	
Rapidly fatal	2 (2.4)	0	2 (3.2)	
Severity of the disease				
Hospital admission	44 (53.7)	12 (63.2)	32 (50.8)	0.434
Oxygen supplement requirement	43 (52.4)	8 (42.1)	35 (55.6)	0.432
Positive pressure ventilation requirement	12 (14.6)	4 (21.1)	8 (12.7)	0.459
Death	1 (1.2)	0	1 (1.6)	1.0
Admission to ICU	0	0	0	NA
Treatment data				
Treatment with a neuraminidase inhibitor	12 (14.6)	12 (63.2)	0	<0.01
Discontinuation of antibiotic treatment	27 (32.9)	4 (21.1)	23 (36.5)	0.271

a COPD, chronic obstructive pulmonary disease; ICU, intensive care unit; NA, not applicable.

b Values are *n* (%) unless otherwise indicated.

The most common symptoms were cough (60.9%, 50/82), fever (58.5%, 48/82), and dyspnea (32.9%, 27/82). Forty-three patients (52.4%, 43/82) had severe respiratory disease and required oxygen supplementation. Forty-four patients (53.7%) were hospitalized with a median of hospital stay of 6 days (interquartile range [IQR], 3 to 9), and 38 patients (46.3%) were discharged from the emergency department.

Among the 82 eligible patients, 19 had a flu-positive diagnostic test result (11 influenza A virus and 8 influenza B virus) (Table 2). There was not a significant statistical difference in demographics and clinical characteristics between patients diagnosed with and those diagnosed without influenza (Table 1).

**TABLE 2:** Results of Xpert Xpress Flu/RSV assay for nasopharyngeal and saliva swabs^*a*^

Microbiological data Xpert Xpress Flu/RSV result	Data by swab type:	
	Nasopharyngeal (*n* = 82)	Saliva (*n* = 82)
Positive influenza PCR (*n* [%])	19 (23.2)	17 (20.7)
Positive influenza A	11 (13.4)	11 (13.4)
Positive influenza B	8 (9.8)	6 (7.3)
Positive RSV PCR (*n*)	0	0
*C_T_* (median [IQR]	31 (21.0-32.0)	33 (23.0-38.0)
Sensitivity (% [95% CI])	NA	89.5 (73.0–100)
Specificity (% [95% CI])	NA	100 (99.2–100)

a RSV, respiratory syncytial virus; *C*_*T*_, cycle threshold; NA, not applicable.

### Comparison of influenza virus detection between saliva and nasopharyngeal swabs.

All study-eligible patients (*n* = 82) had a valid result in their saliva and NP swab samples by the Xpert Xpress Flu/respiratory syncytial virus (RSV) assay (Table 2). There was an overall high agreement (97.6%) between saliva and NP exudate results (80/82; κ = 0.929; 95% confidence interval [CI], 0.832 to 1.0). The detection rate for influenza virus in saliva was lower than that of NP swab (20.7% [17/82] versus 23.2% [19/82]), but a statistical significance was not reached (*P* = 0.5). Only two patients had a discordant result between saliva and NP exudate samples by the Xpert Xpress Flu/RSV assay, detecting influenza B virus in the NP exudate but not in saliva.

Among patients with concordant results, all of them had the same respiratory virus detected (influenza A/B).

The overall sensitivity and specificity for saliva for the diagnosis of confirmed influenza virus was 89.5% (95% CI, 73.0% to 100%) and 100% (95% CI, 99.2% to 100%), respectively.

### Viral load analysis.

*C_T_* values provided by the Xpert Xpress Flu/RSV assay were used as surrogate information for the amount of influenza RNA in the specimens and, therefore, were used to evaluate the viral load.

Among all the patients with the same virus detected in both NP and saliva samples, the *C_T_* values were significantly lower in NP than those in saliva (*P* = 0.001). The median *C_T_* value for NP specimens was 31 (IQR, 21.0 to 32.0), and the median *C_T_* value for saliva specimens was 33 (IQR, 23.0 to 38.0). Overall, a significant correlation between both saliva and NP for the *C_T_* values for influenza virus was observed (*P* = 0.001).

We further analyzed the two discordant cases with a negative PCR result for a saliva swab and positive result for an NP swab. Manual inspection of the amplification curves of the Xpert Xpress Flu/RSV assay in these two patients showed that influenza RNA was amplified from the saliva sample, indicating successful detection of influenza (*C_T_* = 40) despite the machine indicating a negative result.

Among the 82 eligible patients at the emergency department during the 2019 to 2020 influenza season, no cases of respiratory syncytial virus (RSV) were detected by the Xpert Xpress Flu/RSV assay.

## DISCUSSION

Our study shows that saliva collected with a dry swab might be a convenient alternative way for sampling for influenza A and B viruses.

The nasopharyngeal swab is commonly used for the detection of respiratory viruses but is an unpleasant procedure according to observed patient reactions, as they tend to resist it. Moreover, the waiting time for health care providers to collect NP exudates often causes a delay in clinical practice even when using rapid diagnostic assays and can also cause in many cases an infection control risk to health care workers.

We have shown, together with other authors (13–15), that lower respiratory samples obtained from intubated patients have a better diagnostic yield than NP specimens for the diagnosis of influenza and that, if possible, both should be combined. Saliva has been proposed as an alternative sample type for influenza diagnosis (6, 9, 10, 16, 17), but its validity has not been reported by many molecular assays and a sufficient volume is difficult to obtain from the majority of our patients. No studies of the use of saliva swab in adult patients have been reported so far.

We found a high overall agreement between saliva swab and NP exudate samples when tested by Xpert Xpress Flu/RSV assay. There was no significant difference in the detection rate of influenza virus between both samples, and the sensitivity and specificity of the saliva swab were high. This finding shows the utility of saliva swab as a noninvasive diagnostic specimen type for patients admitted to the emergency department of a large tertiary-care hospital.

We observed only two discordant results; two patients had influenza B detected in the NP exudate but not in their saliva swab. However, manual inspection of the amplification curves showed that influenza RNA had been amplified in saliva with high *C_T_*s (*C_T_*, 40) that the test reported as a negative result. Therefore, for patients with high clinical suspicion of influenza infection but a negative saliva result, RNA amplification curves should be reviewed or NP exudate tested. Of note, there was a good correlation in the viral load between paired saliva swab and NP exudate (*P* = 0.001), and most of the patients had a higher viral load in NP than in the saliva swab.

Previous studies tested saliva for 16 different respiratory viruses using multiplex RT-PCR (6) and found adenovirus more frequently in saliva than in NP samples but detected influenza A and rhinovirus more frequently in NP specimens than in saliva. Therefore, they were not able to conclude if one of the sampling methods was consistently more sensitive than the other. The RT-PCR assay that we used also tested for RSV in both types of specimens obtained. Unfortunately, no cases of RSV were detected in our cohort, and we could not evaluate the use of the saliva swab for detection of this virus.

The sensitivity and specificity of saliva swab in this study were higher than those previously found by other authors that used conventional saliva collection with different molecular techniques and included a different patient population (16, 17). However, they were similar to those found by To et al. (10) who also evaluated conventional saliva collection but used the Xpert Xpress Flu/RSV assay as diagnostic test for influenza and enrolled hospitalized adult patients, including those presenting severe disease. Of note, these authors also showed a reduction in cost and time related to the collection of saliva compared with those of NP samples. Early detection of influenza virus is important for guiding antiviral and antibacterial treatment, for infection control and public health measures (9).

There were some limitations to the present study. First, we recruited only adult patients, and therefore, we cannot perform comparisons with other studies that evaluated younger adults and pediatric patients (16). Second, this study included only patients who attended the emergency department with probably more severe diseases and higher viral loads than patients in other settings. Third, the study had to be interrupted because of the arrival of the coronavirus disease 19 (COVID-19) pandemic that did not allow us to reach the sample size expected in our study design. The performance of saliva sampling for COVID testing has already been shown to vary by test platform (12), and this will likely also be true for influenza (and RSV) testing. However, this fact might be mitigated by the use of saliva swab in saline media rather than expectorated saliva.

In conclusion, this study showed that saliva swab might be a feasible clinical specimen to use for detecting influenza virus as well as NP swab when used with highly sensitive diagnostic tests, such as Xpert Xpress Flu/RSV. Saliva swab can be easily self-collected with minimal equipment and discomfort and should be considered a valuable diagnostic specimen type in clinical or research settings.

## MATERIALS AND METHODS

### Study design, participants, and setting.

This was a prospective diagnostic validity study, including adult patients who were admitted to the emergency department of a tertiary-care hospital from January to March 2020. Patients who had a NP specimen collected for detection of presumed influenza infection were eligible. Once the NP specimen and a written informed consent were obtained from the patient, a saliva swab was collected.

Data recorded on the patient included age, sex, date of admission and discharge, flu vaccination, immunodepression status, symptoms at admission, underlying diseases, Charlson’s comorbidity score, McCabe and Jackson score, severity of the disease, microbiological results (PCR), degree of discomfort of sample collection, flu treatment, changes in antibiotic therapy, and contact precautions.

Patients were considered to have a severe disease if they required oxygen supplementation, required positive pressure ventilation, were admitted to the intensive care unit or cardiac intensive care unit, or passed away during the stay at the hospital.

This study was approved by the by the Ethics Committee of Hospital General Universitario Gregorio Marañón (code MICRO.HGUGM.2019-007).

### Specimen collection and laboratory testing.

The saliva specimen was collected by rubbing a dry unflocked swab with no transport medium (Linfkar Healthcare GmbH, Düsseldorf, Germany) on the inside the patient cheeks until the cotton was saturated (minimum of ∼25 seconds). The size of the head of the saliva swab was 1 cm by 0.5 cm. The NP specimen was collected with a flocked swab (Copan Diagnostics, Brescia, Italy) that was inserted into both nostrils of the patient until resistance was felt at the nasopharynx. Then, it was rotated 180 degrees, withdrawn, and transported in 1.5 ml of viral transport medium (UTM viral transport medium; Copan Diagnostics).

NP and saliva swab specimens were transported from the emergency department immediately to the laboratory (30 min maximum) at room temperature. Saliva swabs were resuspended at the laboratory in 200 μl of saline solution, and the entire volume was used for the analysis.

NP and saliva swab specimens were tested for influenza A and influenza B virus using the Xpert Xpress Flu/RSV assay. All amplification curves were inspected manually. NP and saliva specimens were stored at −80°C after the analysis.

### Data analysis.

The comparison of influenza detection rate in saliva and NP samples was assessed by McNemar’s test. Agreement between saliva and NP results was determined using k statistics (18). The sensitivity and specificity of influenza virus detection in the saliva swab samples were calculated using the NP swab specimens as the reference standards. Cycle threshold (*C_T_*) values were compared by using the Wilcoxon signed-rank test. The correlation of *C_T_* values between saliva and NP samples was performed using the Spearman correlation coefficient. A *P* value of <0.05 was required to achieve statistical significance. All statistical analyses were assessed using SPSS Statistics 21, IBM, Chicago, IL, USA.

## References

[1] Sueki A, Matsuda K, Yamaguchi A, Uehara M, Sugano M, Uehara T, Honda T. 2016. Evaluation of saliva as diagnostic materials for influenza virus infection by PCR-based assays. Clin Chim Acta 453:71–74. doi:10.1016/j.cca.2015.12.006. 26656311

[2] Chiarella FC, Culebras E, Fuentes-Ferrer ME, Picazo JJ. 2016. Evaluation of the Alere i Influenza A&B assay for rapid identification of influenza A and influenza B viruses. J Med Microbiol 65:456–461. doi:10.1099/jmm.0.000249. 26967368

[3] Gonzalez-Del Vecchio M, Catalan P, de Egea V, Rodriguez-Borlado A, Martos C, Padilla B, Rodriguez-Sanchez B, Bouza E. 2015. An algorithm to diagnose influenza infection: evaluating the clinical importance and impact on hospital costs of screening with rapid antigen detection tests. Eur J Clin Microbiol Infect Dis 34:1081–1085. doi:10.1007/s10096-015-2328-7. 25620782

[4] Lopez Roa P, Catalan P, Giannella M, Garcia de Viedma D, Sandonis V, Bouza E. 2011. Comparison of real-time RT-PCR, shell vial culture, and conventional cell culture for the detection of the pandemic influenza A (H1N1) in hospitalized patients. Diagn Microbiol Infect Dis 69:428–431. doi:10.1016/j.diagmicrobio.2010.11.007. 21396540

[5] Nguyen Van JC, Camelena F, Dahoun M, Pilmis B, Mizrahi A, Lourtet J, Behillil S, Enouf V, Le Monnier A. 2016. Prospective evaluation of the Alere i Influenza A&B nucleic acid amplification versus Xpert Flu/RSV. Diagn Microbiol Infect Dis 85:19–22. doi:10.1016/j.diagmicrobio.2015.11.012. 26899154

[6] Kim YG, Yun SG, Kim MY, Park K, Cho CH, Yoon SY, Nam MH, Lee CK, Cho YJ, Lim CS. 2017. Comparison between saliva and nasopharyngeal swab specimens for detection of respiratory viruses by multiplex reverse transcription-PCR. J Clin Microbiol 55:226–233. doi:10.1128/JCM.01704-16. 27807150 PMC5228234

[7] Bilder L, Machtei EE, Shenhar Y, Kra-Oz Z, Basis F. 2011. Salivary detection of H1N1 virus: a clinical feasibility investigation. J Dent Res 90:1136–1139. doi:10.1177/0022034511413283. 21700809

[8] Suda Y, Nagatomo M, Yokoyama R, Ohzono M, Aoyama K, Zhang X, Nakajima K, Murakami N, Shinoda T, Hirota T, Yanagihara S, Nishi JI. 2015. Highly sensitive detection of influenza virus in saliva by real-time PCR method using sugar chain-immobilized gold nanoparticles; application to clinical studies. Biotechnol Rep (Amst) 7:64–71. doi:10.1016/j.btre.2015.05.004. 28626716 PMC5466045

[9] To KK, Lu L, Yip CC, Poon RW, Fung AM, Cheng A, Lui DH, Ho DT, Hung IF, Chan KH, Yuen KY. 2017. Additional molecular testing of saliva specimens improves the detection of respiratory viruses. Emerg Microbes Infect 6:e49. doi:10.1038/emi.2017.35. 28588283 PMC5520312

[10] To KKW, Yip CCY, Lai CYW, Wong CKH, Ho DTY, Pang PKP, Ng ACK, Leung KH, Poon RWS, Chan KH, Cheng VCC, Hung IFN, Yuen KY. 2019. Saliva as a diagnostic specimen for testing respiratory virus by a point-of-care molecular assay: a diagnostic validity study. Clin Microbiol Infect 25:372–378. doi:10.1016/j.cmi.2018.06.009. 29906597

[11] Hanson KE, Barker AP, Hillyard DR, Gilmore N, Barrett JW, Orlandi RR, Shakir SM. 2020. Self-collected anterior nasal and saliva specimens versus health care worker-collected nasopharyngeal swabs for the molecular detection of SARS-CoV-2. J Clin Microbiol 58:e01824-20. doi:10.1128/JCM.01824-20. 32817233 PMC7587122

[12] Yee R, Truong TT, Pannaraj PS, Eubanks N, Gai E, Jumarang J, Turner L, Peralta A, Lee Y, Dien Bard J. 2021. Saliva is a promising alternative specimen for the detection of SARS-CoV-2 in children and adults. J Clin Microbiol 59:e02686-20. doi:10.1128/JCM.02686-20. 33239380 PMC8111155

[13] Giannella M, Rodriguez-Sanchez B, Roa PL, Catalan P, Munoz P, Garcia de Viedma D, Bouza E, Gregorio Maranon Task Force for Pneumonia G. 2012. Should lower respiratory tract secretions from intensive care patients be systematically screened for influenza virus during the influenza season? Crit Care 16:R104. doi:10.1186/cc11387. 22697813 PMC3580661

[14] López Roa P, Rodríguez-Sánchez B, Catalán P, Giannella M, Alcalá L, Padilla B, García de Viedma D, García Viedma D, Muñoz P, Bouza E. 2012. Diagnosis of influenza in intensive care units: lower respiratory tract samples are better than nose-throat swabs. Am J Respir Crit Care Med 186:929–930. doi:10.1164/ajrccm.186.9.929. 23118087

[15] Rello J, Pop-Vicas A. 2009. Clinical review: primary influenza viral pneumonia. Crit Care 13:235. doi:10.1186/cc8183. 20085663 PMC2811908

[16] Robinson JL, Lee BE, Kothapalli S, Craig WR, Fox JD. 2008. Use of throat swab or saliva specimens for detection of respiratory viruses in children. Clin Infect Dis 46:e61–e64. doi:10.1086/529386. 18444806 PMC7107872

[17] Yoon J, Yun SG, Nam J, Choi SH, Lim CS. 2017. The use of saliva specimens for detection of influenza A and B viruses by rapid influenza diagnostic tests. J Virol Methods 243:15–19. doi:10.1016/j.jviromet.2017.01.013. 28111058

[18] Landis JR, Koch GG. 1977. The measurement of observer agreement for categorical data. Biometrics 33:159–174. doi:10.2307/2529310. 843571

